# Vascular narrowing in pulmonary arterial hypertension is heterogeneous: rethinking resistance

**DOI:** 10.14814/phy2.13159

**Published:** 2017-03-21

**Authors:** Nina Rol, Esther M. Timmer, Theo J.C. Faes, Anton Vonk Noordegraaf, Katrien Grünberg, Harm‐Jan Bogaard, Nico Westerhof

**Affiliations:** ^1^ Department of Pulmonary DiseasesVU University Medical CenterAmsterdamthe Netherlands; ^2^ Laboratory for PhysiologyInstitute for Cardiovascular ResearchICaR‐VUVU University Medical CenterAmsterdamthe Netherlands; ^3^ Department of Physics and Medical TechnologyVU University Medical CenterAmsterdamthe Netherlands; ^4^ Department of PathologyRadboud UMCNijmegenthe Netherlands

**Keywords:** External diameters, internal diameters, pulmonary vascular resistance, resistance vessels, wall thickness

## Abstract

In idiopathic pulmonary arterial hypertension (PAH), increased pulmonary vascular resistance is associated with structural narrowing of small (resistance) vessels and increased vascular tone. Current information on pulmonary vascular remodeling is mostly limited to averaged increases in wall thickness, but information on number of vessels affected and internal diameter decreases for vessels of different sizes is limited. Our aim was to quantify numbers of affected vessels and their internal diameter decrease for differently sized vessels in PAH in comparison with non‐PAH patients. Internal and external diameters of transversally cut vessels were measured in five control subjects and six PAH patients. Resistance vessels were classified in Strahler orders, internal diameters 13 *μ*m (order 1) to 500 *μ*m (order 8). The number fraction, that is, percentage of affected vessels, and the internal diameter fraction, that is, percentage diameter of normal diameter, were calculated. In PAH, not all resistance vessels are affected. The number fraction is about 30%, that is, 70% of vessels have diameters not different from vessels of control subjects. Within each order, the decrease in diameter of affected vessels is variable with an averaged diameter fraction of 50–70%. Narrowing of resistance vessels is heterogeneous: not all vessels are narrowed, and the decrease in internal diameters, even within a single order, vary largely. This heterogeneous narrowing alone cannot explain the large resistance increase in PAH. We suggest that rarefaction could be an important contributor to the hemodynamic changes.

## Introduction

Idiopathic pulmonary arterial hypertension (iPAH) is generally assumed to result from decreased internal diameters of the small vessels, often labeled as resistance vessels. Resistance may increase up to a factor ~4–5 and consequently mean pulmonary artery pressure may increase by a similar amount (Chazova et al. 1995; Lankhaar et al. 2008, 2006; Overbeek et al. 2008; Palevsky et al. 1989; Peacock et al. 2011; Stacher et al. 2012). Internal diameters of these resistance vessels can be reduced by vasoconstriction or (concentric) remodeling, and because in only a minority of PAH patients acute vasodilator challenges result in a substantial pressure decrease, concentric remodeling is considered to be the major factor in vessel narrowing and augmentation of pulmonary vascular resistance (Palevsky et al. 1989). Inhibition of vascular remodeling is considered as an effective therapeutic target.

However, quantitative information on remodeling of resistance vessels from iPAH patients, in comparison with non‐PAH, is almost exclusively limited to the increase in relative wall thickness, expressed as WT = (*d*_*o*_–*d*_*i*_)/*d*_*o*_^* *^× 100%, with *d*_*o*_ and *d*_*i*_ external and internal diameter. In general, wall thickness is most often presented in terms of average values, not discriminating between vessel size (Palevsky et al. 1989; Stacher et al. 2012). Chazova et al. (1995) reported on averaged wall thickening in six diameter groups of arteries, and showed that the wall thickness increase in iPAH, and therefore the internal diameter change, may depend on vessel size. In the same study, wall thickening of the pulmonary veins is also reported. Anderson et al. (1973) reported similar findings in three PAH patients. However, only averaged data were reported, while the percentage of vessels affected and the possible variation in degree of vessels narrowing were not. Hence, to the best of our knowledge, in the published literature on PAH, very little quantitative information has been provided pertaining to the number of affected vessels and the degree of internal diameter decreases of differently sized vessels in the (peripheral) pulmonary vascular bed. Quantitative information on internal diameters is required to accurately estimate vascular resistance.

Therefore, we determined internal and external diameters of pulmonary resistance vessels (13 to 500 *μ*m; Strahler orders 1–8) and number of occluded vessels in histological slides of healthy subjects and PAH patients, and quantified the fraction of narrowed vessels and their degree of narrowing in each Strahler order (Horsfield 1978; Huang et al. 1996, 2011).

## Methods

### Internal and external diameters

**Figure 1:**
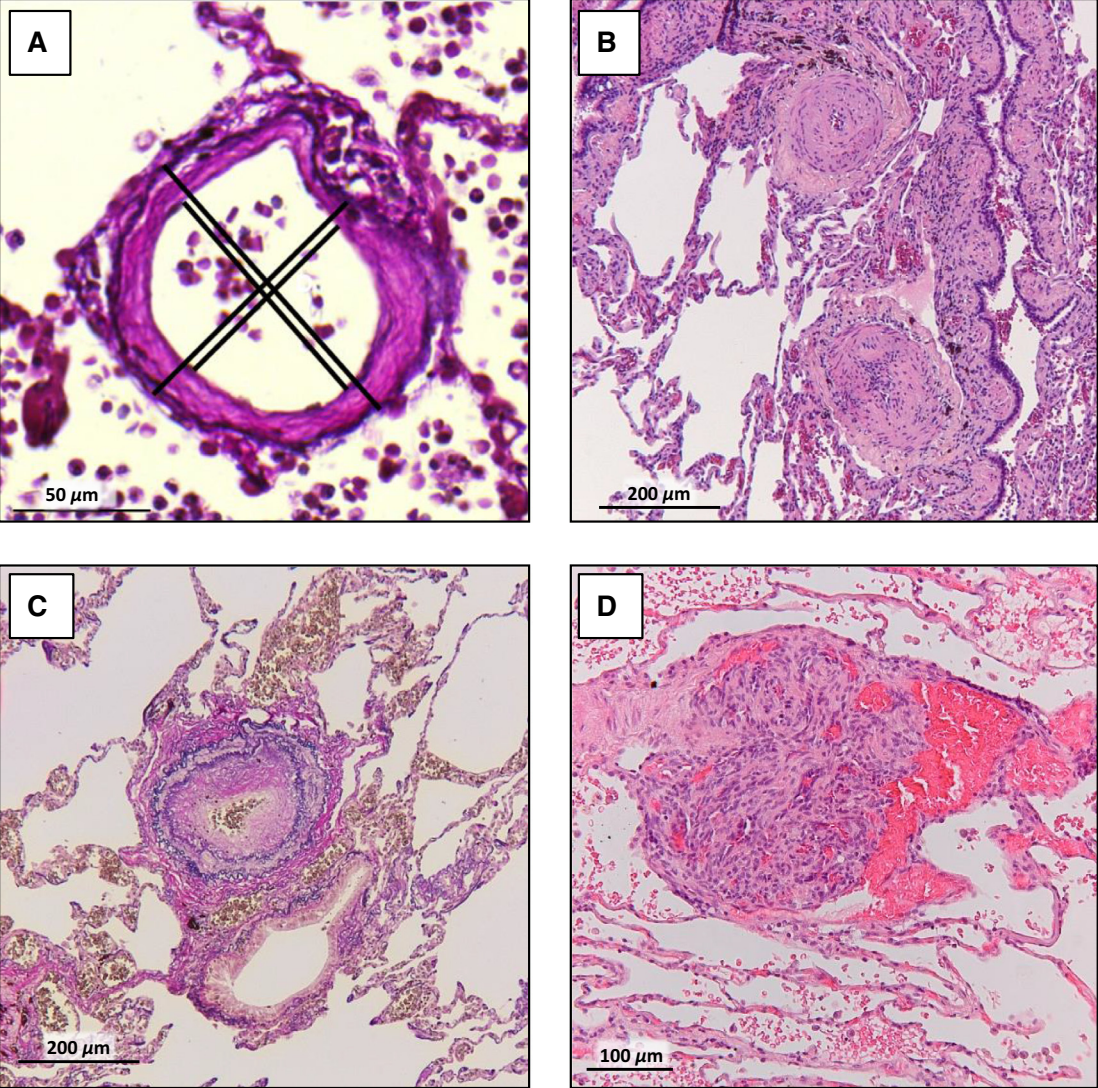
Wall thickness measurement and vascular remodeling seen in PAH. (A) Example of determining the wall thickness of a pulmonary vessel by measuring the lamina elastica externa and lumen in two perpendicular directions in all transversally cut vessels between 13 and 500 *μ*m encountered per slide. Examples of typical vascular remodeling observed in the lung tissue of pulmonary arterial hypertension patients in our study group, like intimal fibrosis (B; H&E staining) and medial thickening (C; Elastica van Gieson staining) in a pulmonary artery and a plexiform lesion (D; H&E staining). Scalebar indicates 100 *μ*m.

Lung tissue was obtained from the biobank at the department of pathology of the VU University Medical Center, Amsterdam, the Netherlands. The study was approved by the Institutional Review Board on Research Involving Human Subjects of the VU University Medical Center. Tissue of control subjects was obtained from people who died acutely due to traumatic causes (mean age: 40.8 (24–79) years; 100% male). Tissues were analyzed from five idiopathic PAH and one hereditary PAH patient (#2), all of whom were diagnosed following prevailing diagnostic criteria (three males, all normal BMI, four nonsmokers). Diagnosis was confirmed at autopsy by histological observation of vascular remodeling and plexiform lesions (Fig. 1). Mean age was 54 (45–57) years at death. The patient cases were part of an earlier study (Overbeek et al. 2009). Lung parenchyma was perfused with and fixed in formalin and tissue from different lobes was embedded in paraffin. Serial sections of 4 *μ*m thickness were subjected to conventional hematoxylin and eosin staining to confirm diagnosis and Elastica van Gieson staining for evaluation of structural changes. Per subject, the external and internal diameter of all transversally cut vessels (arterial and venous, grouped) encountered in one tissue block with average size of 2.75 cm^2 ^± 0.26 cm^2^ between 13 and 500 *μ*m were measured, by determining the mean distance between the lamina elastica externa and lumen in two perpendicular directions (see Fig. 1A). Number of vessels counted in each Strahler order is reported in the Table S1.

By comparing PAH internal diameters with their normal values found in the controls, the number of narrowed PAH vessels and their degree of narrowing were determined. This was done per order for Strahler orders *m *=* *1 to *m *=* *8, that is, resistance vessels with diameters of 13 *μ*m to 500 *μ*m (Table S1).(Huang et al. 1996, 2011) We introduced for each order *m*, a number fraction *F*_*n*_(*m*), which is the fraction of narrowed vessels with respect to the total number of vessels, and a diameter fraction *F*_*d*_(*m*), which is the internal diameter as a fraction of the normal internal diameter. The diameter fraction *F*_*d*_(*m*) of a narrowed vessel thus expresses the percentage that is left of a narrowed vessel. On both number and diameter fraction, linear regression analysis was performed.

### Determinations of number fraction and diameter fraction

To determine *F*_*n*_(*m*) and *F*_*d*_(*m*), firstly, the internal diameters *d*_*i*_ were plotted as a function of the external diameters *d*_*o*_. Subsequently, linear regression analysis was performed on the logarithmic diameter values of all control data to get a control relation between internal and external diameter for normal vessels. Furthermore, the iPAH data were categorized in orders based on external diameter, as follows: the categorization criteria were obtained from Horsfield, who labeled vessels based on internal diameter (Horsfield 1978; Huang et al. 1996). These internal diameters were converted to external diameters using our control relation.

Next, a one‐sided 90% prediction interval for the control relation, called the prediction line, was determined to define normal and narrowed vessels in iPAH. Narrowed vessels were the iPAH vessels lying below this prediction line. In every order *m*, the number of narrowed vessels was counted and divided by the total number of vessels, to calculate the number fraction *F*_*n*_(*m*). Linear regression analysis was performed on *F*_*n*_(*m*) to construct a regression line of *F*_*n*_(*m*) as a function of order *m* with a 95% confidence interval.

To calculate one value of the diameter fractions *F*_*d*_(*m*) for each order *m*, first, the normal internal diameters of each narrowed vessel were obtained from the control relation. Second, their measured internal diameter was divided by their calculated normal internal diameter to calculate the diameter fraction of each narrowed vessel, and these were plotted per order. Next, linear regression analysis was performed on the diameter fractions to construct a regression line of the diameter fraction *F*_*d*_(*m*) as a function of order *m* with a 95% confidence interval.

### Occluded vessels

Immunofluorescent staining with von Willebrand factor antibody conjugated to FITC (vWf, 1:200 dilution, overnight incubation at 4°C, Abcam, Ab8822) combined with *α*‐smooth muscle actin conjugated to Cy3 (*α*‐SMA, 1:200 dilution, 2 h at room temperature, Sigma, C6198) was performed on control and PAH lung tissue to determine the number of occluded vessels as percentage of the total number of vessels. Approximately, 30–68 vessels below 100 *μ*m were studied in 7–12 fields of view per subject. Occlusion was defined by vWf staining completely comprising the luminal surface of a vessel. Imaging was performed at 10× magnification with an Axiovert 200 Marianas inverted wide‐field fluorescence microscope (Carl Zeiss Microscopy, Jena, Germany). These percentages were averaged for controls and patients and given as mean^ ^± SEM.

## Results

Patient characteristics, including hemodynamic profiles, of the PAH patients are, when known, shown in Table 1.

**Table 1:** General patient characteristics

Male/female, no	3/3
Age at death (years)	54 (45–57)
mPAP (mmHg)	59 (44–76)
PAWP (mmHg)	6 (0–11)
PVR (dyns·cm^‐5^)	856.6 (455–1587)
CO (L/min)	4.8 (3.6–5.8)
CI (L/min/m^2^)	2.6 (1.9–3.5)
Smoking history (never/current/former, n)	4/0/2
Therapy	
Prostacyclin	5
ERA	1
PDE‐5 inhibitor	1
ABS	2

ABS, atrial balloon septostomy; CI, cardiac index; CO, cardiac output; ERA, endothelin receptor antagonist; mPAP, mean pulmonary artery pressure; PAWP, pulmonary artery wedge pressure; PDE‐5, phosphodiesterase 5; PVR, pulmonary vascular resistance.

### Internal and external diameters

**Figure 2:**
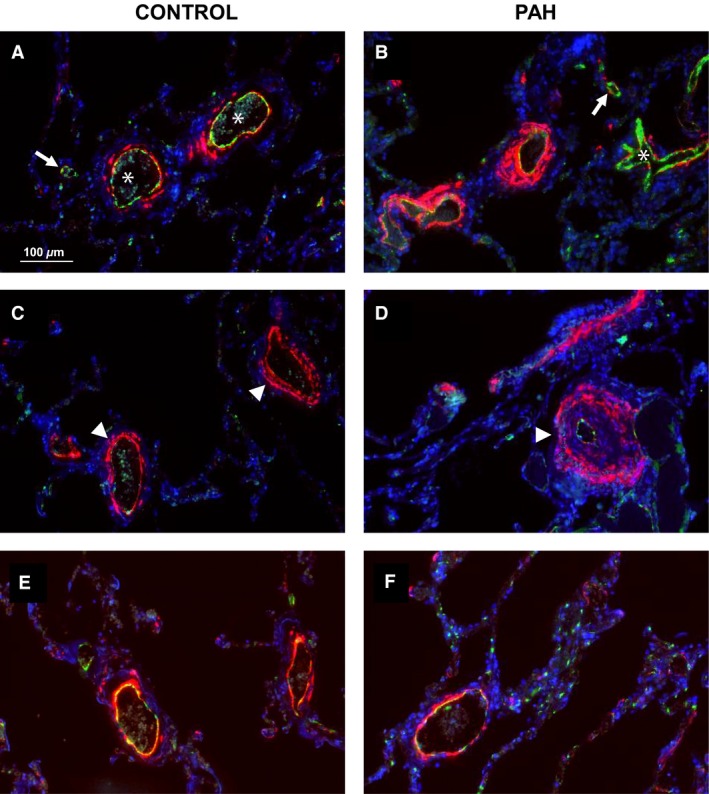
Vascular occlusions assessed with immunofluorescent staining. Besides thickening of the intimal (B, asterix) and medial (D, arrowhead) vascular layer in pulmonary arterial hypertension compared with control (A, asterisks; C, arrowhead), a great number of vessels is not occluded (A and D, arrows) when assessed with immunofluorescent von Willebrand factor staining. Vascular remodeling does not occur in all vessels in PAH tissue (E) and still look similar to control vessels (F). Scale bar indicates 100 *μ*m.

Heterogeneous vascular remodeling was seen in PAH patients, with varying thickening of the intimal and medial vascular layers. PAH patients showed intimal fibrosis and medial thickening but no significant increase in complete vascular occlusions; 10.4 ± 2.3% of the vessels of PAH patients were occluded, compared to 6.6 ± 3.5% in control subjects (Fig. 2).

**Figure 3:**
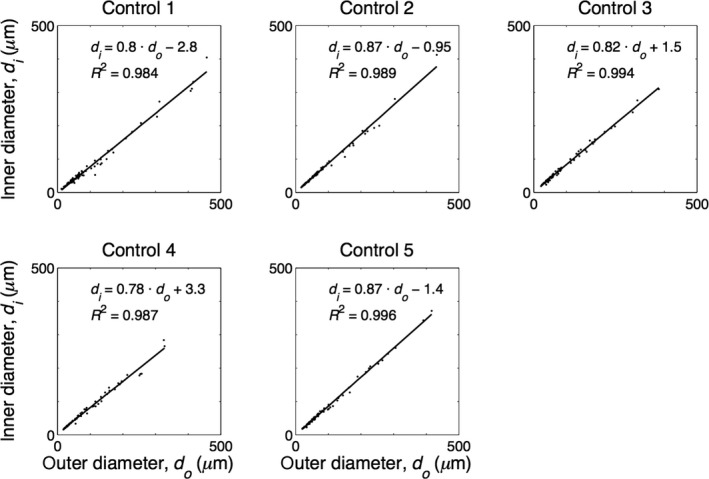
Internal diameter (*d*i) as a function of external diameter (*d*o) as measured in five control subjects. Regression line is depicted with black line.

**Figure 4:**
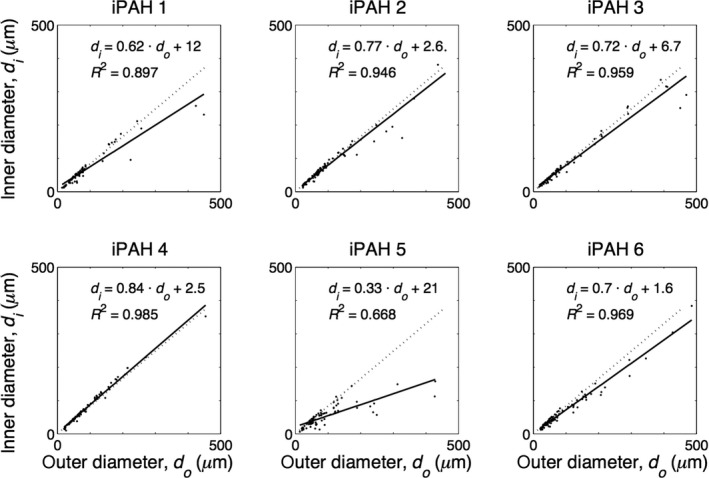
Internal diameter (*d*i) as a function of external diameter (*d*o) as measured in six PAH patients. Regression line, depicted with black line, and the averaged control regression line (all control data of taken together, dotted line): *d*_i_ = 0.824 *d*_o_ + 0.260, *R* ^2^ = 0.993.

Figures 3, 4 show the relations between internal and external diameters of the control subjects and PAH patients, respectively, with their linear fits. The control data showed a clear linear relation (*R* ^2^ between 0.984 and 0.996). The mean relative wall thickness (WT) of all control vessels was about 16% (mean^ ^± 95% CI: 16.3%^ ^± 0.7%). Patient data showed a weaker relation between internal and external diameter (*R* ^2^ between 0.668 and 0.985) and a mean WT of all vessels of about 22% (22.0%^ ^± 1.2%). However, many PAH data points appeared to follow the control regression line indicating unaffected vessels.

**Figure 5:**
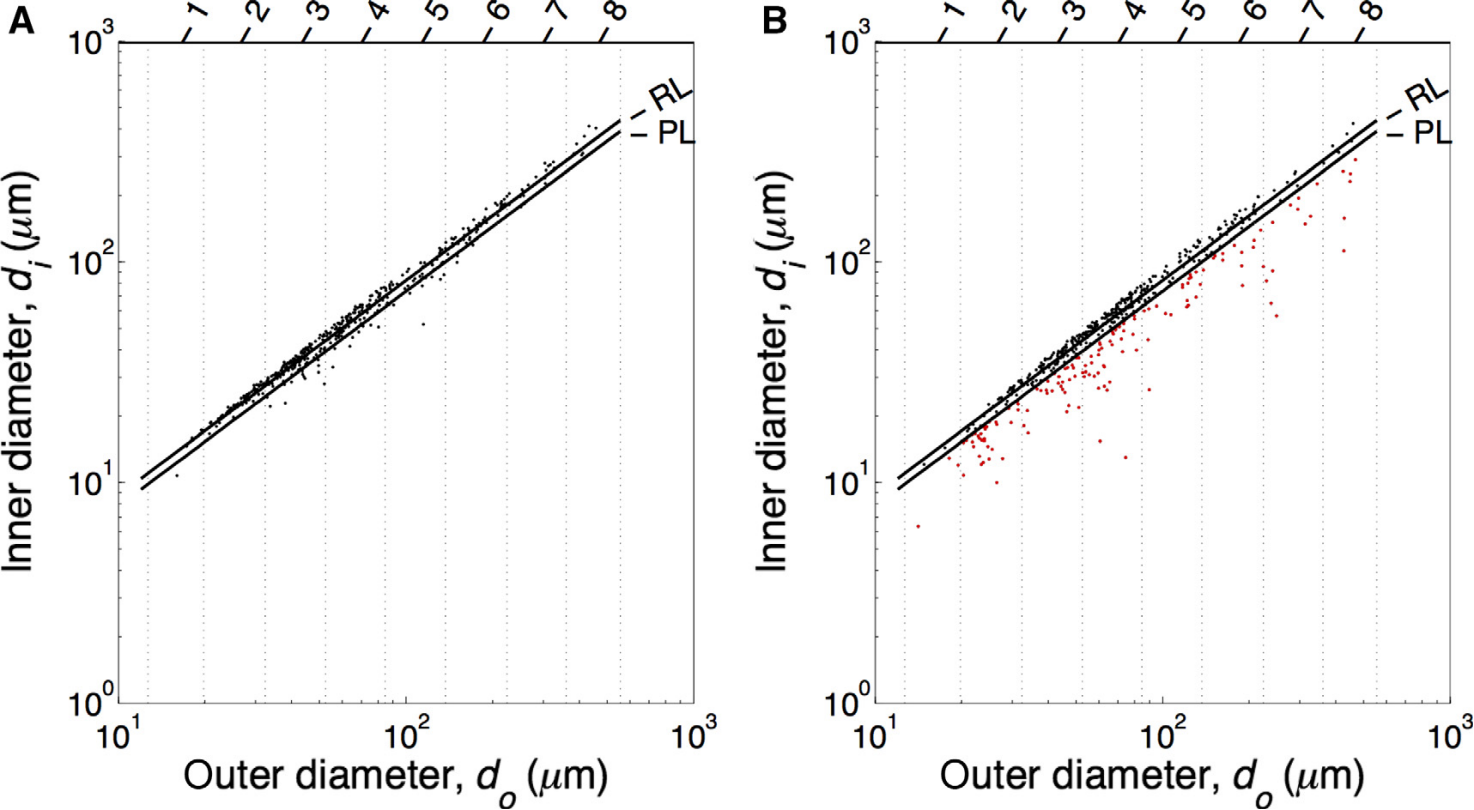
Log–log plots of the diameters of control (A) and PAH vessels. Diameters of control (A) and PAH (B) vessels per order (Strahler order numbers indicated on top), both with the regression line (RL) and the lower 90% prediction line (PL) of the control data. The vertical lines indicate the order limits. The PAH data points under the control PL are classified as narrowed vessels (red dots).

Figure 5 shows the diameter relations on a log–log scale. Diagram A shows the data of all controls with the control regression line and the lower 90% control prediction line. Panel B shows the data of all PAH patients with the control regression line and lower 90% control prediction line. About 70% of the PAH data points are above this prediction line and are considered as vessels with a nonreduced internal diameter, while 30% (in gray) are below the prediction line and thus have a significantly reduced inner diameter. The normal vessels had a mean WT of 14% ± 0.6%, and the narrowed vessels had a mean WT of 39% ± 2%.

**Figure 6:**
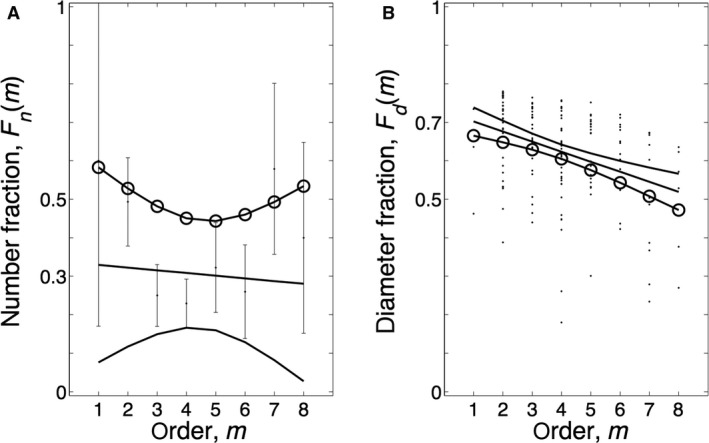
Number and diameter fractions per order. (A) Number fractions *F*_n_(m) as function of order. (B) Diameter fractions of the vessels *F*_d_(m) per order. The regression lines with their 95% confidence intervals are shown. The slope of *F*_n_(m) is not significant (*P *=* *0.70); the slope of *F*_d_(m) is significant (*P *<* *0.0001). The circles on the upper and lower confidence limits indicate the values of *F*_n_(m) and *F*_d_(m) that predict the worst case that a maximal number of vessels are involved with a minimal inner diameter, thereby contributing maximally to the resistance increase.

The number fractions *F*_*n*_(*m*) (diagram A) and the diameter fractions (diagram B) of the PAH vessels are shown by order in Figure 6. The linear regression lines of the data with the 95% CIs are shown.

**Figure 7:**
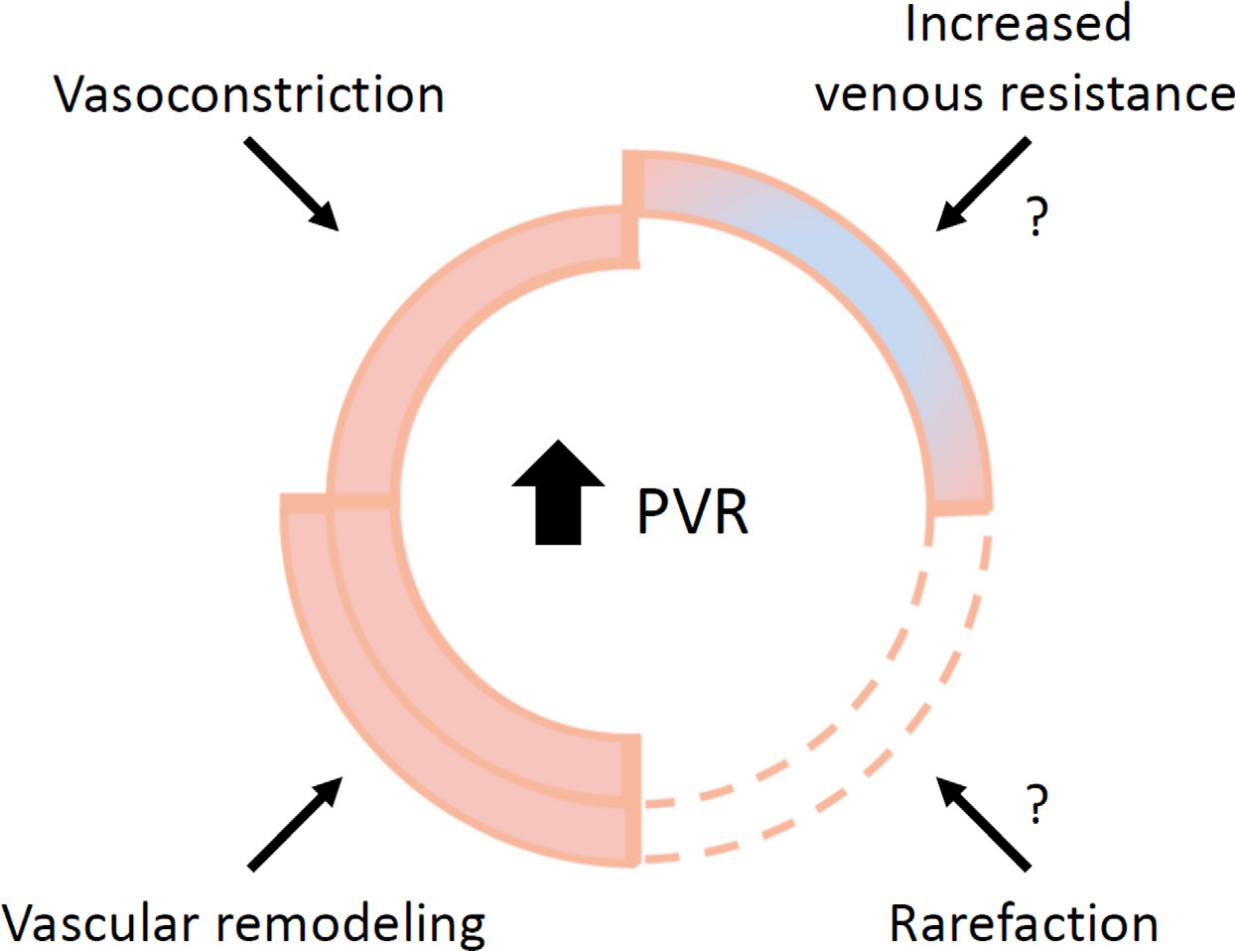
Contributors to increased pulmonary vascular resistance. Increased pulmonary vascular resistance (PVR) can not only be attributed to vasoconstriction and pulmonary vascular remodeling; it is likely that increased venous resistance and rarefaction also contribute to hemodynamic changes seen in pulmonary arterial hypertension.

## Discussion

We determined external and internal diameters of pulmonary vessels in the range of 13–500 *μ*m, Strahler orders 1–8. The internal and external diameters of the control vessels were all located in a narrow range around the regression line (Fig. 5A), while in the PAH patients, on average, about 30% of the vessels were narrowed (Fig. 6A, number fraction), thus ~70% was in the range of the control subjects. This was the first heterogeneity observed: not all vessels are narrowed. A second heterogeneity was that in each Strahler order, the affected vessels showed a large range of variation in internal diameter.

### Diameter and wall thickness

We here report data on internal and external diameters of the pulmonary vasculature, and thus were able to derive relative wall thickness from the slope of the relation between internal and external diameters (Fig. 3, 4) as WT = 1‐slope. Only wall thickness (intima plus media) was studied, changes in wall composition (e.g., isolated media hypertrophy etc.) were not determined. To the best of our knowledge, number and diameter fractions, presented in Figure 6, implying heterogeneous narrowing, are unique and a direct comparison with the literature is not possible.

In control subjects, we find an average slope of 0.83, thus an average wall thickness of ~17%, which is close to wall thickness reported by Chazova (~18%) (Chazova et al. 1995). (Stacher et al. 2012) however, recently reported considerably thicker vessel walls in controls (about 30%). Of all vessels studied, 8.3% was occluded. Because vWF stains both, endothelial cells and platelets, our histological analysis does not allow for a discrimination between occlusion due to thrombotic material and occlusion due to endothelial hyperproliferation.

In PAH, we found the number of occluded vessels to be similar to control. Diameter relations vary greatly, but their average slope (Fig. 4) (when all vessels are included) is 0.66, resulting in an averaged wall thickness of 34%. Calculation of averaged wall thickness including the occluded vessels would only minimally increase the calculated average wall thickness, to about 39%. Chazova et al. (1995) report, at systolic/diastolic PAP 120/60 mmHg, thus estimated mPAP ~70 mmHg, a wall thickness of 50% for vessels with diameters between 25 and 250 *μ*m. Stacher et al. (2012) report wall thickness in hypertension at a mPAP ~58 mmHg about 60%. However, it is not entirely clear if all vessels are included in the Chazova et al. (1995) and Stacher et al. (2012) studies. Palevsky et al. (1989) use wall area, with WA = 100 × wall area/total vessel cross‐sectional area and found 64%, and thus wall thickness equaling √(1‐fractional WA) is about 60% with range 40–75%, at a mPAP of ~60 mmHg.

### Can vascular changes predict pulmonary vascular resistance?

Calculation of PVR, to estimate the contribution of vascular remodeling to resistance increase, requires the determination of the total number and internal diameters of all vessels in the entire lung. These data are not available. However, a relative increase in PVR from control to PAH can be obtained as follows. We found a number fraction of 30% (Fig. 6A), that is, 70% of normal vessels remain. When we assume that, in controls and PAH patients, the percentage of occluded vessels are similar, that length changes do not occur and that all narrowed vessels are completely closed (i.e., thus not contributing to flow; this leads to an overestimation of the actual resistance), 30% of abnormal vessels would result in an 1/0.7 = 1.4‐fold increase in PVR in PAH. Maximal PVR in healthy individuals, within the limits of normal is 99 dynes·cm^‐5^.(Peacock et al. 2011) Thus, the value of 1.499 = 138.6 dynes·cm^‐5^, is far lower than the resistance in our patient group, which is 857 dynes cm^‐5^ (Table 1). This can partly be explained by the vascular reservoir capacity and blood flow recruitment the lung has, strengthened by the finding of Burrowes et al. (2011) who calculated that more than 50% of the vessels have to be obstructed to increase PVR.

The number of occluded vessels we found and the small number of affected vessels between control and PAH, is considerably smaller than the 65% of mechanical obstruction that Burrowes et al. (2011) calculated to be required to increase the mPAP above 25 mmHg.

The relatively large number of vessels with the same diameter as control vessels, if still functional, would suggest considerable effect of vasodilation. Since this is not the case, the vessels with normal wall thickness are either not functioning normally or pulmonary resistance is greatly affected by rarefaction or (changed) venous resistance.

Rarefaction has been debated (Mooi and Wagenvoort 1983). In animal experiments, the resistance of the venous system has been shown to be considerable (Hakim and Kelly 1989). An increased capillary pressure has been shown in patients with PAH, also suggesting a high venous resistance (Kafi et al. 1998). Recently Dorfmuller et al. (2014) showed venous involvement in chronic thromboembolic pulmonary hypertension. We therefore suggest that rarefaction and increased venous resistance should be studied in future research to determine their role in hemodynamic changes.

### Limitations

Although studying pulmonary pathology in human tissue gives valuable insides, technical drawbacks are: Tissue blocks used in this study were retrospectively selected from the tissue biobank stored for diagnostic purposes. We randomly selected tissue blocks from unknown locations, but it cannot be guaranteed to be representative for the entire lung. That could also be true for the selection of rounded, transversally cut vessels. Also, longitudinal consistency of changes in the pulmonary vasculature has not been shown and could add another dimension of heterogeneity in vascular remodeling in PAH. Location of occlusive lesions in the pulmonary vasculature (proximal vs. distal) could have a different impact on PVR. Studies on three‐dimensional vessel analyses and of the pulmonary vasculature below 25 *μ*m could improve understanding of the relation between vascular remodeling and PVR.

Diameters have been determined using samples of autopsy material where vessels are possibly (maximally) vasodilated and are therefore not necessarily representative of the in vivo situation. The possibility of differences in vasoactive state at death and potential differences of changes in vasodilation/constriction during fixation are, at present, not known. However, there is no reason to assume differences regarding vasoconstriction and vasodilation between the control and PAH vessels after fixation and embedding.

Whether or not rarefaction of vessels exists could not be determined. To assess this phenomenon, lengths and diameters and the total number of vessels or total endothelial surface area in control and PAH patients should be measured and compared in various regions of the lungs.

The presented data suggest that vascular remodeling results in a relatively small increase in PVR, which is insufficient to fully explain pulmonary vascular resistance as observed in PAH patients. Absolute numbers of vessels, wall thickness measurements of vessels below 25 *μ*m, and even information on the venous vasculature are required.

## Conclusion

We show that structural changes in the pulmonary vasculature of PAH patients are heterogeneous: 70% of vessels are not altered and of the affected vasculature, the degree of diameter decrease varies greatly. These diameter changes alone cannot explain the resistance increase in PAH.

## Perspectives

It is generally assumed that in pulmonary arterial hypertension (PAH), structural changes of the (small) pulmonary arteries in combination with vasoconstriction are the predominant cause of the increased resistance to blood flow and high pressure. We show that structural changes in the pulmonary arterial vasculature are heterogeneous: only 30–50% of the resistance arteries are narrowed and narrowing varies in vessel orders and possibly along vessel length. These changes cannot fully explain the resistance increase in PAH. Future studies should investigate increases in wall thickness of veins, wall thickness differences along the length of resistance vessels, and arterial/venous rarefaction to provide better estimates of pulmonary vascular resistance.

## Conflict of Interest

No relations with industry for all authors.

## Data Accessibility

## Supporting information




**Table S1:** Supplementary material.
