# Supplementary material for: Vascular narrowing in pulmonary arterial hypertension is heterogeneous: rethinking resistance
Source: Physiol Rep. 2017 Mar 21;5(6):e13159. doi: 10.14814/phy2.13159 (PMC5371554; doi:10.14814/phy2.13159)
Supplement: Supplementary file 1 — Table S1: Supplementary material. [file PHY2-5-e13159-s001.docx]

|  | Number of measurements | |
| --- | --- | --- |
| Strahler order | Control  (n = 5) | PAH  (n = 6) |
| 1 | 7 | 5 |
| 2 | 71 | 73 |
| 3 | 120 | 110 |
| 4 | 101 | 170 |
| 5 | 44 | 62 |
| 6 | 43 | 48 |
| 7 | 21 | 19 |
| 8 | 8 | 15 |
